# A Thermodynamic Model of Monovalent Cation Homeostasis in the Yeast *Saccharomyces cerevisiae*

**DOI:** 10.1371/journal.pcbi.1004703

**Published:** 2016-01-27

**Authors:** Susanne Gerber, Martina Fröhlich, Hella Lichtenberg-Fraté, Sergey Shabala, Lana Shabala, Edda Klipp

**Affiliations:** 1 Faculty of Biology, Johannes Gutenberg-Universität Mainz, Germany; 2 Theoretical Biophysics, Humboldt-Universität zu Berlin, Germany; 3 Babraham Institute, Cambridge, United Kingdom; 4 Molecular Bioenergetics, University of Bonn, Bonn, Germany; 5 School of Land and Food, University of Tasmania, Hobart, Australia; University of Tokyo, JAPAN

## Abstract

Cationic and heavy metal toxicity is involved in a substantial number of diseases in mammals and crop plants. Therefore, the understanding of tightly regulated transporter activities, as well as conceiving the interplay of regulatory mechanisms, is of substantial interest. A generalized thermodynamic description is developed for the complex interplay of the plasma membrane ion transporters, membrane potential and the consumption of energy for maintaining and restoring specific intracellular cation concentrations. This concept is applied to the homeostasis of cation concentrations in the yeast cells of *S*. *cerevisiae*. The thermodynamic approach allows to model passive ion fluxes driven by the electrochemical potential differences, but also primary or secondary active transport processes driven by the inter- play of different ions (symport, antiport) or by ATP consumption (ATPases). The model—confronted with experimental data—reproduces the experimentally observed potassium and proton fluxes induced by the external stimuli KCl and glucose. The estimated phenomenological constants combine kinetic parameters and transport coefficients. These are in good agreement with the biological understanding of the transporters thus providing a better understanding of the control exerted by the coupled fluxes. The model predicts the flux of additional ion species, like e.g. chloride, as a potential candidate for counterbalancing positive charges. Furthermore, the effect of a second KCl stimulus is simulated, predicting a reduced cellular response for cells that were first exposed to a high KCl stimulus compared to cells pretreated with a mild KCl stimulus. By describing the generalized forces that are responsible for a given flow, the model provides information and suggestions for new experiments. Furthermore, it can be extended to other systems such as e.g. *Candida albicans*, or selected plant cells.

## Author Summary

Metals, and particularly their positively charged ions (cations), are an integral part of our environment, and all living organisms are exposed to metals in their natural habitat. Even though significant efforts have already been made by experimental and theoretical analysis of the individual components of transport systems and individual transport-mechanisms, such efforts did not result in an integration of the highly connected and complex system. The development of kinetic networks might well contribute to the understanding and visualization of cation homeostasis. However, such kinetic systemic analysis would require more detailed biochemical information than is currently available. We circumvented this problem by using an entirely phenomenological approach of the theory of non-equilibrium thermodynamics. The methodology does not require the detailed understanding of structure, function or kinetic parameters of individual constituents of the system but produces some unique parameters related to thermodynamic couplings between different ion fluxes and ATP consumption. These estimated phenomenological constants combine the kinetic parameters and transport coefficients and control the coupling of fluxes. The model predictions are in good agreement with the biological understanding of the roles of the transporter proteins. Our modeling approach might contribute to the development of new diagnostic and therapeutic purposes with cation-homeostasis as key-target.

## Introduction

System responses to cation induced stress play a pivotal role in a wide range of essential cellular processes. A major challenge for the cell is to maintain optimum cytoplasmic concentrations of cations even under rapidly changing external conditions and perturbations such as salt, osmotic, or alkaline pH stress. The alkali metals such as sodium, potassium (or lithium) are considered as vitally important co-factors for a variety of enzymatic reactions and for structural and functional roles in cell metabolism [1,2]. However, they are also potent toxic pollutants at high concentrations and relevant for severe biological and medical phenomena (i.e. blocking of functional groups on important bio-molecules as well as denaturation of enzymes and DNA damage)[3] [4].

**Fig 1:**
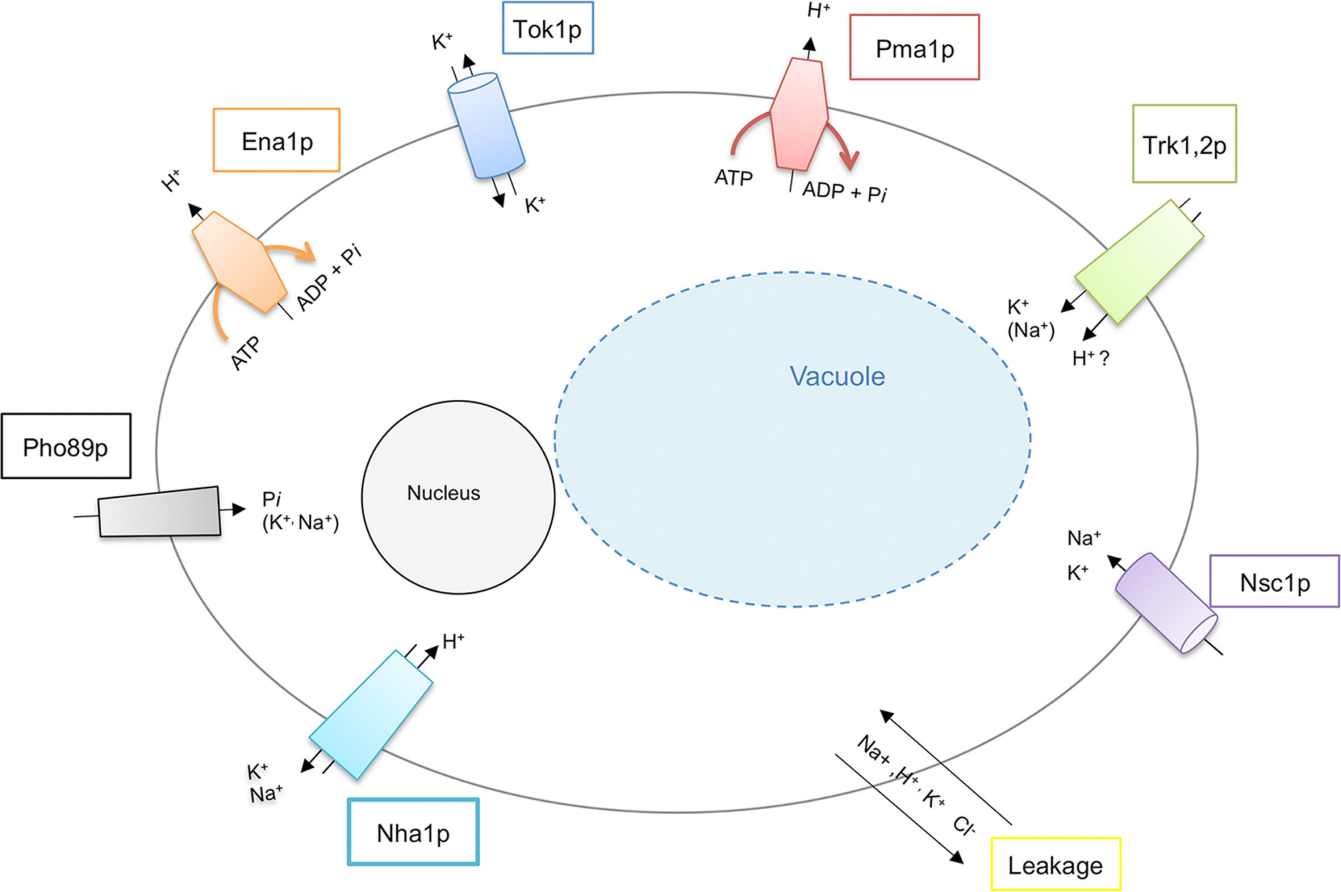
Sketch of the system and the model-relevant key elements.

For the unicellular eukaryote *Saccharomyces cerevisiae* most of the proteins responsible for uptake and extrusion of sodium, potassium, protons and chloride across the cellular membrane have been identified (see Fig 1) and some transport mechanisms are well described (see Table 1 and [5,6] [7]). However, despite considerable experimental work and some modeling efforts [8,9] the integration of transport systems to ensure homeostasis and the interplay between particular ion transport proteins and factors controlling the rate of transport are not fully understood. Filling this gap could positively affect a wide area of application: Geo- and natural sciences, as well as agronomists consider the issue under the aspects of environmental pollution caused by extensive use of some (heavy) metals and metal compounds as e.g. in fungicides and disinfectants. Related agricultural research concerned the ability of plants to tolerate or adapt to a range of environmental stress conditions like e.g. aridity or very high or almost nil concentrations of salt. In biomedical sciences ion homeostasis receives increasing attention due to its role in a number of pathological conditions, such as a variety of neurodegenerative diseases, metabolic disorders and malignant transformations [10] [11]. Therefore, the understanding of tightly regulated transporter activities and the interplay of regulatory mechanisms is of substantial interest and could contribute to the developments in plant growing sciences or to improvements regarding food safety. Furthermore, a better understanding could influence the development of new treatments for fungal infections or the design of new pharmacological agents to treat neurodegenerative diseases.

**Table 1:** Overview of transmembrane ion transporters and channels.

Name	Type	Substrate specificity	Main function
Trk1,2	Uniporter	K^+^ (Rb^+^)	K^+^—uptake
Tok1	Channel	K^+^	K^+^—extrusion
Nsc1	Channel	unspecific	Function unknown
Ena1	ATPase	Na^+^, Li^+^ (K^+^, Rb^+^)	Detoxification
Nha1	Antiporter	Na^+^, K^+^ (Li^+^, Rb^+^), H^+^	Na^+^-, K^+^-Extrusion
Pma1	ATPase	H^+^	H^+^-Extrusion
Pho89	Symporter	P_i_ (Na^+^, K^+^)	Phosphate uptake

We suggest a predictive mathematical model to gain better understanding of the principles of homeostasis employed by nature.

The regulation of intracellular cation content is an important and complex cellular task. In comparison to the relatively controlled environment of most animal cells (within the tissue context), single-celled organisms like e.g. some algae and fungi must tolerate a wide range of sometimes rapidly changing environmental conditions such as osmotic pressures, pH, or salt concentrations in their natural habitats. Moreover, yeast cells accumulate potassium from relatively dilute solutions to sustain a cytosolic K^+^ concentration within the range of approximately 175–300 mM to counterbalance the intracellular high negative charge from proteins as well as inorganic and organic negatively charged polyanions [6,12].

Besides supporting a stable and balanced intracellular cation content, monovalent cation transport is also required for other physiological functions such as maintenance of the cell volume and internal pH, the membrane potential, protein synthesis, and enzyme activation [13–15]. To ensure viability even under adverse external environmental conditions yeast has evolved several response systems to saline, osmotic and alkaline pH stress [12,16,17]. To maintain an optimum cytoplasmic pH of about 6.5 and a stable balanced intracellular sodium/potassium ratio yeast cells invest high amounts of biological energy through ATP hydrolysis and employ three distinct strategies [5]: i) strict discrimination between alkali metal cations at the level of influx (e.g. higher affinity of transporters for potassium than for sodium), ii) proper disposal of toxic cations and iii) selective sequestration of cations in organelles. Eight transport proteins relevant to the regulation and maintenance of intracellular alkali-metal cation content are well characterized (see Table 1). Comprehensive reviews detail further specifics and mechanisms, regulatory elements and the "long-term" regulation processes by transcription [5,6,12].

The paper is organized as follows: We first introduce a general thermodynamic concept for the description and analysis of cellular cation fluxes and concentrations. Second, we assign specific parameters, which were obtained from experiments with starved yeast cells. We then use the experimental data for parameter estimation and present model simulations predictive for scenarios not used for parameterization.

The underlying experimental scenario is as follows: Yeast cells are starved overnight in water to lose all mobile nutrients and cations. Fluxes are measured from time 0. At time 300 s the cells are exposed to defined concentrations of KCl (0.01–10 mM). At time 600 s, glucose is added. Potassium and proton fluxes are measured with specific electrodes *via* the MIFE method (see Materials and Methods).

## Results

### Thermodynamic derivation of flux calculations

Our description of ion fluxes and their mutual dependencies is based on the concepts of Non-Equilibrium Thermodynamics (NET). Since decades various theories and mathematical descriptions of active and passive transport executed by transmembrane proteins have been developed. These approaches are as different as complex and have already been extensively published [18–22] to mention just a few of them. The classical studies of ion fluxes (e.g. on nerves) have mainly focused on the measurement of the relation between currents and voltage and on the modeling of fluxes caused by combination of single transport systems [23–28]. Typically, every channel or transporter is described with an expression for its current as a function of the membrane potential and the actual concentration of the respective ion. These rate expressions are based on the assumption of linear force-flux relationships, yielding, however, non-linear relations between ion concentrations and ion fluxes.

When modeling the behavior of living cells, the selective description of individual channels/transporters carries the risk of overlooking other ion transport processes by transporters that are not yet characterized or known transporters that have additional functions (e.g. non-specific transport) or membrane leakage. Thus, understanding the system’s behavior requires the integrative investigation of all transport processes, in addition to exploring individual transporters. Many features change simultaneously and should be integrated into a global model in order to obtain a comprehensive picture of the underlying physical processes. This includes transient pH, enzyme activities, cytosolic buffer capacities, chemical reactions, and changes in membrane potential or concentrations of other important ions. However, due to the complexity of the problem and the sparseness of data, typical kinetic network models that describe every reaction and transport step in detail are not yet feasible.

The concept of Non-Equilibrium Thermodynamics, deployed to derive individual transport expressions, provides also a theoretical background to correlate driving forces and the resulting fluxes in cellular systems in a formal manner independent of specific kinetic or statistical models. The relevant forces are the differences in the electrochemical potential of the cations and reaction affinities of biochemical reactions. Fluxes are the resulting fluxes of cations in or out of the cell and the rates of biochemical reactions, respectively. All these irreversible processes lead to a production of entropy. The entropy production for a cellular system can be characterized by the entropy production density [29,30]

$$\sigma ={\vec{J}}_{Q}\text{grad}\left(\frac{1}{T}\right)-\sum_{i=1}^{n_{f}}{\vec{J}}_{i}^{f}\text{grad}\left(\frac{\eta_{i}}{T}\right)+\sum_{i=1}^{n_{r}}J_{i}^{r}\frac{A_{i}}{T}$$ (1)

where *σ* denotes the local entropy production density, *T* is the temperature, ${\vec{J}}_{Q}$ is the heat flow density, ${\vec{J}}_{i}^{f}$ is the diffusion density of component *i*, *η*_*i*_ is the electrochemical potential of component *i*, $J_{i}^{r}$ is the rate of reaction *i*, *A*_*i*_ is the affinity of reaction *i*, and *n*_*f*_ and *n*_*r*_ are the numbers of compounds and reactions, respectively.

The various flows and forces are not independent of each other. A temperature gradient could, for example, induce the diffusion flux of a chemical compound besides the heat flux. Due to the constant temperature in the considered experiments, we can disregard temperature gradients and heat flux in the following reasoning. We take generalized forces as *X*_*j*_. The fluxes are in general non-linear functions of these forces. However, at equilibrium all forces and fluxes vanish. Only in vicinity to equilibrium we can express the fluxes as linear combinations of all forces, based on a Taylor expansion until first order terms as follows:

$$J_{i}^{f,r}=\sum_{j=1}^{n_{f}+n_{r}}\frac{\partial J_{i}}{\partial X_{j}}X_{j}=\sum_{j=1}^{n_{f}+n_{r}}L_{ij}X_{j}$$ (2)

The partial derivatives of the fluxes with respect to the forces are called phenomenological coefficients and will be denoted with *L*_*ij*_. The *L*_*ii*_ are referred to as the "straight coefficients" since they relate the flow *J*_*i*_ to its conjugate driving force *X*_*i*_, in the analogy with either Ohm‘s or Fick‘s laws. The "cross coefficients" *L*_*ij*_, with *j* ≠ *i*, reflect to which extent the flux of species *i* is affected by the non-conjugate forces, *X*_*j*_, in the system. The phenomenological coefficients have to fulfill a number of conditions. Since in the absence of other forces, a single force induces a positive conjugate flux, it holds:

$$L_{ii}\geq 0\;\left(\text{for all}\;i\right)$$ (3)

The fact that the dissipation function is positive implies further that

$$L_{ij}=L_{ji},$$ (4)

which is also known as “Onsager’s reciprocity relation” [31,32], and that

$$\text{Det}\left[L_{ij}\right]\geq 0.$$ (5)

In the following we specify the relevant forces and fluxes for ion transport and biochemical reactions in the considered experiments. In general, these phenomenological coefficients combine kinetic parameters and transport coefficients and are functions of the parameters of the system but are independent of the flows and forces. Once determined from experimental data they provide an informative basis on the control exerted by the coupled fluxes. Specifically interesting for the maintenance of the intracellular cation concentration is the thermodynamic coupling of the individual fluxes. This enables that a flux may occur without or even against its conjugate thermodynamic driving force, which may be a gradient of the electrochemical potential or reaction affinity.

For the cellular response of starved yeast cells to the addition of KCl and glucose we considered the forces resulting from the electrochemical gradients of protons, K^+^, Na^+^, Cl^-^, denoted as grad *η*_H_, grad *η*_K_, grad *η*_Na_, and grad *η*_Cl_, respectively, as well as the affinity *A*_Ar_ of the reactions converting ATP into ADP or reverse. The conjugated fluxes are the fluxes of protons, *J*_H_, potassium, *J*_K_, sodium, *J*_Na_, and chloride, *J*_Cl_, as well as the conversion of ATP to ADP or back, *J*_Ar_. This resulted in the following phenomenological equation system:

$$\begin{array}{l} J_{\text{H}}=-L_{\text{HH}}\text{grad}\left(\frac{\eta_{\text{H}}}{T}\right)-L_{\text{HK}}\text{grad}\left(\frac{\eta_{\text{K}}}{T}\right)-L_{\text{HNa}}\text{grad}\left(\frac{\eta_{\text{Na}}}{T}\right)-L_{\text{HCl}}\text{grad}\left(\frac{\eta_{\text{Cl}}}{T}\right)+L_{\text{HAr}}\frac{A_{\text{Ar}}}{T}\\ J_{\text{K}}=-L_{\text{KH}}\text{grad}\left(\frac{\eta_{\text{H}}}{T}\right)-L_{\text{KK}}\text{grad}\left(\frac{\eta_{\text{K}}}{T}\right)-L_{\text{KNa}}\text{grad}\left(\frac{\eta_{\text{Na}}}{T}\right)-L_{\text{KCl}}\text{grad}\left(\frac{\eta_{\text{Cl}}}{T}\right)+L_{\text{KAr}}\frac{A_{\text{Ar}}}{T}\\ J_{\text{Na}}=-L_{\text{NaH}}\text{grad}\left(\frac{\eta_{\text{H}}}{T}\right)-L_{\text{NaK}}\text{grad}\left(\frac{\eta_{\text{K}}}{T}\right)-L_{\text{NaNa}}\text{grad}\left(\frac{\eta_{\text{Na}}}{T}\right)-L_{\text{NaCl}}\text{grad}\left(\frac{\eta_{\text{Cl}}}{T}\right)+L_{\text{NaAr}}\frac{A_{\text{Ar}}}{T}\\ J_{\text{Cl}}=-L_{\text{ClH}}\text{grad}\left(\frac{\eta_{\text{H}}}{T}\right)-L_{\text{ClK}}\text{grad}\left(\frac{\eta_{\text{K}}}{T}\right)-L_{\text{ClNa}}\text{grad}\left(\frac{\eta_{\text{Na}}}{T}\right)-L_{\text{ClCl}}\text{grad}\left(\frac{\eta_{\text{Cl}}}{T}\right)+L_{\text{ClAr}}\frac{A_{\text{Ar}}}{T}\\ J_{\text{Ar}}=-L_{\text{ArH}}\text{grad}\left(\frac{\eta_{\text{H}}}{T}\right)-L_{\text{ArK}}\text{grad}\left(\frac{\eta_{\text{K}}}{T}\right)-L_{\text{ArNa}}\text{grad}\left(\frac{\eta_{\text{Na}}}{T}\right)-L_{\text{ArCl}}\text{grad}\left(\frac{\eta_{\text{Cl}}}{T}\right)+L_{\text{ArAr}}\frac{A_{\text{Ar}}}{T} \end{array}$$ (6)

Next, we replaced the electrochemical potentials with the expression

$$\eta_{i}=\mu_{i}^{0}+RT\;\text{ln}\;c_{i}+z_{i}F\varphi \;\left(i\in \left\{\text{H,K,Na,Cl}\right\}\right)$$ (7)

with *c*_*i*_ being the ion concentrations and *z*_*i*_ being their charge number, *F* is Faraday‘s constant and *φ* is the membrane potential.

Since we assume homogeneity of concentrations inside and outside of the cell, the gradient of *η*_*i*_ refers to the derivative of *η*_*i*_ with respect to the spatial direction normal to the cell surface. We approximated it with the difference of *η*_*i*_ between cellular environment (out, “*o*”) and cytoplasm (in, “*i*”), i.e. $\Delta \eta_{i}=\eta_{i}^{o}-\eta_{i}^{i}$.

Combined, these considerations resulted in the following equation system:

$$\begin{array}{l} J_{\text{H}}=R\left(L_{\text{HH}}\;\text{ln}\frac{c_{\text{H}}^{i}}{c_{\text{H}}^{o}}+L_{\text{HK}}\;\text{ln}\frac{c_{\text{K}}^{i}}{c_{\text{K}}^{o}}+L_{\text{HNa}}\;\text{ln}\frac{c_{\text{Na}}^{i}}{c_{\text{Na}}^{o}}+L_{\text{HCl}}\;\text{ln}\frac{c_{\text{Cl}}^{i}}{c_{\text{Cl}}^{o}}\right)+\frac{F}{T}\Delta \varphi \left(L_{\text{HH}}+L_{\text{HK}}+L_{\text{HNa}}-L_{\text{HCl}}\right)+L_{\text{HAr}}\frac{A_{\text{Ar}}}{T}\\ J_{\text{K}}=R\left(L_{\text{KH}}\;\text{ln}\frac{c_{\text{H}}^{i}}{c_{\text{H}}^{o}}+L_{\text{KK}}\;\text{ln}\frac{c_{\text{K}}^{i}}{c_{\text{K}}^{o}}+L_{\text{KNa}}\;\text{ln}\frac{c_{\text{Na}}^{i}}{c_{\text{Na}}^{o}}+L_{\text{KCl}}\;\text{ln}\frac{c_{\text{Cl}}^{i}}{c_{\text{Cl}}^{o}}\right)+\frac{F}{T}\Delta \varphi \left(L_{\text{KH}}+L_{\text{KK}}+L_{\text{KNa}}-L_{\text{KCl}}\right)+L_{\text{KAr}}\frac{A_{\text{Ar}}}{T}\\ J_{\text{Na}}=R\left(L_{\text{NaH}}\;\text{ln}\frac{c_{\text{H}}^{i}}{c_{\text{H}}^{o}}+L_{\text{NaK}}\;\text{ln}\frac{c_{\text{K}}^{i}}{c_{\text{K}}^{o}}+L_{\text{NaNa}}\;\text{ln}\frac{c_{\text{Na}}^{i}}{c_{\text{Na}}^{o}}+L_{\text{NaCl}}\;\text{ln}\frac{c_{\text{Cl}}^{i}}{c_{\text{Cl}}^{o}}\right)+\frac{F}{T}\Delta \varphi \left(L_{\text{NaH}}+L_{\text{NaK}}+L_{\text{NaNa}}-L_{\text{NaCl}}\right)+L_{\text{NaAr}}\frac{A_{\text{Ar}}}{T}\\ J_{\text{Cl}}=R\left(L_{\text{ClH}}\;\text{ln}\frac{c_{\text{H}}^{i}}{c_{\text{H}}^{o}}+L_{\text{ClK}}\;\text{ln}\frac{c_{\text{K}}^{i}}{c_{\text{K}}^{o}}+L_{\text{ClNa}}\;\text{ln}\frac{c_{\text{Na}}^{i}}{c_{\text{Na}}^{o}}+L_{\text{ClCl}}\;\text{ln}\frac{c_{\text{Cl}}^{i}}{c_{\text{Cl}}^{o}}\right)+\frac{F}{T}\Delta \varphi \left(L_{\text{ClH}}+L_{\text{ClK}}+L_{\text{ClNa}}-L_{\text{ClCl}}\right)+L_{\text{ClAr}}\frac{A_{\text{Ar}}}{T}\\ J_{\text{Ar}}=R\left(L_{\text{ArH}}\;\text{ln}\frac{c_{\text{H}}^{i}}{c_{\text{H}}^{o}}+L_{\text{ArK}}\;\text{ln}\frac{c_{\text{K}}^{i}}{c_{\text{K}}^{o}}+L_{\text{ArNa}}\;\text{ln}\frac{c_{\text{Na}}^{i}}{c_{\text{Na}}^{o}}+L_{\text{ArCl}}\;\text{ln}\frac{c_{\text{Cl}}^{i}}{c_{\text{Cl}}^{o}}\right)+\frac{F}{T}\Delta \varphi \left(L_{\text{ArH}}+L_{\text{ArK}}+L_{\text{ArNa}}-L_{\text{ArCl}}\right)+L_{\text{ArAr}}\frac{A_{\text{Ar}}}{T} \end{array}$$ (8)

The fluxes are considered as outward directed, i.e. $J_{i}=J_{i}^{i\to 0}$and membrane potential difference is Δ*φ* = *φ* ^*i*^ − *φ* ^*o*^.

#### Internal and external concentration changes

The resulting internal and external concentration changes of the ions were calculated as:

$$\begin{array}{l} \frac{\text{d}}{\text{d}t}c_{\text{H}}^{o}=\frac{J_{\text{H}}\cdot Surf}{V_{out}}\;\frac{\text{d}}{\text{d}t}c_{\text{H}}^{i}=-\frac{J_{\text{H}}\cdot Surf}{V_{in}}\\ \frac{\text{d}}{\text{d}t}c_{\text{K}}^{o}=\frac{J_{\text{K}}\cdot Surf}{V_{out}}\;\frac{\text{d}}{\text{d}t}c_{\text{K}}^{i}=-\frac{J_{\text{K}}\cdot Surf}{V_{in}}\\ \frac{\text{d}}{\text{d}t}c_{\text{Na}}^{o}=\frac{J_{\text{Na}}\cdot Surf}{V_{out}}\;\frac{\text{d}}{\text{d}t}c_{\text{Na}}^{i}=-\frac{J_{\text{Na}}\cdot Surf}{V_{in}}\\ \frac{\text{d}}{\text{d}t}c_{\text{Cl}}^{o}=\frac{J_{\text{Cl}}\cdot Surf}{V_{out}}\;\frac{\text{d}}{\text{d}t}c_{\text{H}}^{i}=-\frac{J_{\text{H}}\cdot Surf}{V_{in}} \end{array}$$ (9)

where *Surf* is the cellular surface, (*V*_*in*_) is the internal volume added up over all cells of the system, and (*V*_*out*_) is the volume of the extracellular compartment.

We followed this general description of concentration changes with two exceptions accounting for the specific experimental conditions. First, we assumed that the internal pH is buffered and the internal proton concentration changes with the proton flux as follows:

$$\frac{\text{d}}{\text{d}t}c_{\text{H}}^{i}=-\frac{J_{\text{H}}\cdot Surf}{V_{i}}\cdot Bf$$ (10)

In principle, *Bf* is a function depending on the pH, but it can be approximated by a constant for a wide range of intracellular pH values. This modification is equivalent to the proton buffering function as introduced before [8,33].

Second, the change of the ATP concentration *c*_*ATP*_ was calculated using the following equation:

$$\frac{\text{d}}{\text{d}t}c_{\text{ATP}}=k_{\text{ATP}incr}-k_{\text{ATP}decr}\cdot c_{\text{ATP}}$$ (11)

where *k*_ATPincr_ and *k*_ATPdecr_ are kinetic constants. *k*_ATPdecr_ was calculated based on the value of *k*_ATPincr_ and the assumed maximal value of ATP after stimulation, *ATP*_*stimulus*_, as *k*_ATP*decr*_ = *k*_ATP*incr*_ / *ATP*_*stimulus*_. Since cells have been starved before the beginning of the experiment, we set *k*_ATPincr_ to 0 before glucose addition and estimated its value from the experimental data after the glucose pulse.

The reaction affinity is

$$A_{\text{Ar}}=\frac{RT}{{\bar{c}}_{\text{ATP}}}\left(c_{\text{ATP}}-{\bar{c}}_{\text{ATP}}\right)\left(1+K_{eq}\right)$$ (12)

with ${\bar{c}}_{\text{ATP}}$ the equilibrium concentration of ATP, *K*_*eq*_ the equilibrium constant of the reaction and *R* being the gas constant (for a detailed derivation see [34]).

We also considered that ATPases change their substrate affinity after glucose addition [35,36] and thus allowed for a change of the values of the respective phenomenological coefficients.

As an example, a change in the coefficient for ATP driven H^+^ export was calculated as

$$\frac{\text{d}}{\text{d}t}L_{\text{HAr}}=k_{incr\text{HAr}}-k_{decr\text{HAr}}\cdot L_{\text{HAr}}$$ (13)

with *k*_incrHAr_ and *k*_decrHAr_ being the parameters for the increase and the decrease of the value for *L*_HAr_. *k*_decrHAr_ was calculated based on the value of *k*_incrHAr_ and an estimated maximal value of *L*_HAr_, *L*_HAraG,_, by *k*_*decr*HAr_ = *k*_*incr*HAr_ / *L*_HAraG_. The coefficients *L*_HH_, *L*_KK_, and *L*_KAr_ were calculated accordingly.

Finally, the dynamics of the membrane potential have been calculated from the relevant ion fluxes as follows

$$\frac{\text{d}}{\text{d}t}\Delta \varphi =-\frac{2F}{C_{m}}\left(J_{\text{H}}+J_{\text{K}}+J_{\text{Na}}-J_{\text{Cl}}\right)$$ (14)

with *C*_*m*_ being the membrane capacitance [37]. The set of Eqs (7–14) constitutes the general thermodynamic model for the fluxes of protons, sodium, potassium, and chloride in the presence of hydrolysable ATP.

#### Connection between phenomenological coefficients and individual membrane transport proteins

The approach applied here aims to construct a model truly representing ion fluxes without modeling each transporter in full detail. Therefore, the phenomenological coefficients present lumped contributions of different active and passive transport processes as well as leakage. The set of known active transporters or channels related to the considered coefficients are listed in Table 2. We also briefly summarize the known function of the various transporters to guide the potential interpretation of the parameter estimation and simulation results presented below.

**Table 2:** Assignment of phenomenological coefficients to their realizing transporters.

Phenomenological Coefficients	Potential contribution of the transporters
*L*_HH_	Pma1p, Trk1/2p, Nha1p, Leakage
*L*_HK_	Nha1p, Trk1/2p (if H^+^/K^+^ symport)
*L*_HNa_	Nha1p, Trk1/2p (if H^+^/Na^+^ symport)
*L*_HAr_	Pma1p
*L*_HCl_	H^+^/Cl^-^ symporter
*L*_KK_	Tok1p, Trk1/2p, Pho89p, Nsc1p, Leakage
*L*_KAr_	Inward directed K^+^-ATPase
*L*_NaNa_	Trk1/2p, Pho89p, Nsc1p, Leakage
*L*_ClCl_	Cl^-^ -leakage, Trk1/2p, H^+^/Cl^-^-symporter

- Proton transport appears to be strictly coupled to transport of K^+^, Na^+^, or Cl^-^ or to ATP consumption. The respective proteins are Nha1p, Trk1,2p, a potential H^+^/Cl^-^ symporter and the Pma1p. Beyond leakage no specific proton channel is identified yet.
- Transport of Na^+^ and Cl^-^ is only coupled to H^+^ transport, presumably *via* Nha1p, a potential H^+^/Cl^-^ symporter.
- The active sodium transporter Ena1 is only expressed to relevant amounts upon salt or pH stress, but not under our experimental conditions [6], thus no Na^+^/ATP coupling was considered.
- K^+^ transport can occur independently via Tok1p or coupled to proton transport via Nha1p and possibly Trk1,2p. There is no K^+^-ATPase for yeast systems reported in the literature.

By estimating the phenomenological coefficients using experimental data, knowledge can be gained about the individual transporters contributing to them.

### Simulation results

**Fig 2:**
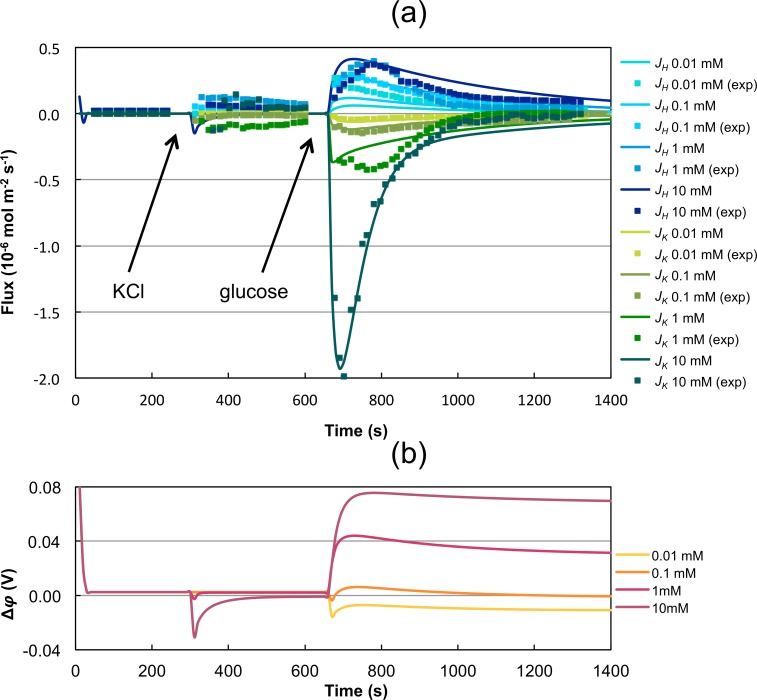
Simulations using model M1 with parameter set P1. Data resulting from microelectrode ion flux measurements (MIFE) of potassium fluxes (green) and proton fluxes (blue) in the *S*. *cerevisiae* wild-type strain PLY232 [43] due to four different stimuli with KCl (0.01 mM, 0.1 mM, 1 mM, 10 mM) at time 300 s followed by addition of glucose at time 600 s (S1 Data). Simulations were performed using the model with parameter set P1. a) Presentation of the best fit (solid lines) and the experimental data (squares), b) prediction of the membrane potential.

The generalized thermodynamic description was developed for the complex interaction of specific cation plasma membrane transporters, the membrane potential, and the consumption of energy for maintaining and restoring the respective intracellular cation concentrations based on the theory of NET. The model was then challenged with experimental data representing independent measurements of potassium and proton fluxes (Fig 2A) in *S*. *cerevisiae* wild type strains after treatment with four different concentrations of KCl followed by addition of glucose (S1 Data). The phenomenological coefficients were estimated to define the degree of coupling between the considered ion fluxes as well as the rate of ATP/ADP conversion. The dynamics of the phenomenological coefficients as well as a basic sensitivity analysis can be found in the S1 Text.

Below we discuss two model variants and their biological interpretation.

#### Model M1: Model with K^+^-importing ATPase

In a first step the model was fitted to the data by including all phenomenological coefficients listed in Table 2 and restricting them by Eqs. 3–5. Parameter set P1 (see Table 3) reproducing the data best favors an ATP-driven K^+^-import, indicating the existence of a K^+^-ATPase. This model showed the best—in the sense of being closest to the data—result (Fig 2A). However, it was assumed to be artificial since first, the membrane potential obtained positive values after glucose addition (Fig 2B) and second the K^+^ influx and H^+^ efflux would be completely independent of the proton-pumping ATPase Pma1. Instead, H^+^ would be driven out of the cell depending on the preceding active transport of K^+^ into the cells. Furthermore, a K^+^-importing ATPase has not yet been identified in the plasma membrane of *S*. *cerevisiae*. Although the existence of such an ATPase cannot completely be ruled out, we disregard the mechanism resulting from those model parameters. For more information regarding phenomenological coefficients and ATPases see [38–40]

**Table 3:** Initial concentrations, global quantities and volumes, and estimated parameters for P1. Estimated model parameters for stress with 4 different concentrations of KCl. All other *L*s could be set to 0 without affecting the goodness of fit.

**Global quantities and volumes**	**Value**	**Source**
*V*_*in*_	1.8· 10^−11^ m^3^	Calculation
*V*_*out*_	2.85 · 10^−6^ m^3^	Exp. Condition
*T*	296 K	Exp. Condition
*F*	96,485 C/mol	Faraday constant
*Surf* (of all cells)	2.29 · 10^−5^ m^2^	Calculation
Proton buffer capacity (*pbc*)	200mM pH	Experimental observation
Conversion factor (*cf*)	1000 mM/M	
*K*	0.01136	estimated
*C*_*ATP*_	0.07255 mM	estimated
Δ*φ*	0.0742 V	estimated
**Initial conditions**	**Values**	**Source**
*H*_*out*_	3.162 ·10^−3^	Exp. condition (pH 5.5)
*K*_*out*_	0.1 mM	Exp. condition
*Cl*_*out*_	0.1 mM	Exp. condition
*ATP*	2.303 mM	estimated between 0 and 2.5 mM
*ATP*_*stimulus*_	2.5 mM	Özalp et al. [65]
*KCl*_*stimulus*_	0.01, 0.1, 1, 10 mM	Exp. condition
pH_*in*_	5.528	estimated between 5 and 7
*K*_*in*_	90.39 mM	estimated between 60 and 100 mM
*Cl*_*in*_	8.217 mM	estimated between 0.1 and 10 mM
*Na*_*in*_	5.778 mM	estimated between 5 and 30 mM
*Na*_*out*_	0.0863 mM	estimated between 0.01 and 0.1 mM
**Phenomenological and stoichiometric coefficients**	**Parameter values**	**Source**
*L*_HH*init*_	2.64 · 10^−8^ mol^2^/(J· m^2^ · s)	estimated
*L*_HHaG_	2.68 · 10^−8^ mol^2^/(J· m^2^ · s)	estimated
*L*_HNa_	-1.03 · 10^−8^ mol^2^/(J· m^2^ · s)	estimated
*L*_HAraG_	4.09 · 10^−10^ mol^2^/(J· m^2^ · s)	estimated
*L*_HCl_	1.33 · 10^−9^ mol^2^/(J· m^2^ · s)	estimated
*L*_KKinit_	4.05 · 10^−22^ mol^2^/(J· m^2^ · s)	estimated
*L*_KKaG_	2.91 · 10^−4^ mol^2^/(J· m^2^ · s)	estimated
*L*_KAraG_	-1.26 · 10^−4^ mol^2^/(J· m^2^ · s)	estimated
*L*_NaNa_	8.74 · 10^−9^ mol^2^/(J· m^2^ · s)	estimated
*L*_ClCl_	3.49 · 10^−7^ mol^2^/(J· m^2^ · s)	estimated
*k*_*incr*HH_	3.51 · 10^−7^ mol^2^/(J· m^2^ · s^2^)	estimated
*k*_*incr*HAr_	2.39 · 10^−9^ mol^2^/(J· m^2^ · s^2^)	estimated
*k*_*incr*KK_	5.15 · 10^−9^ mol^2^/(J· m^2^ · s^2^)	estimated
*k*_*incr*KAr_	-9.93 · 10^−10^ mol^2^/(J· m^2^ · s^2^)	estimated
*k*_ATP*incr*_	0.0991 mol/(m^3^ · s)	estimated

#### Model M2: Model without K^+^-ATPase

**Fig 3:**
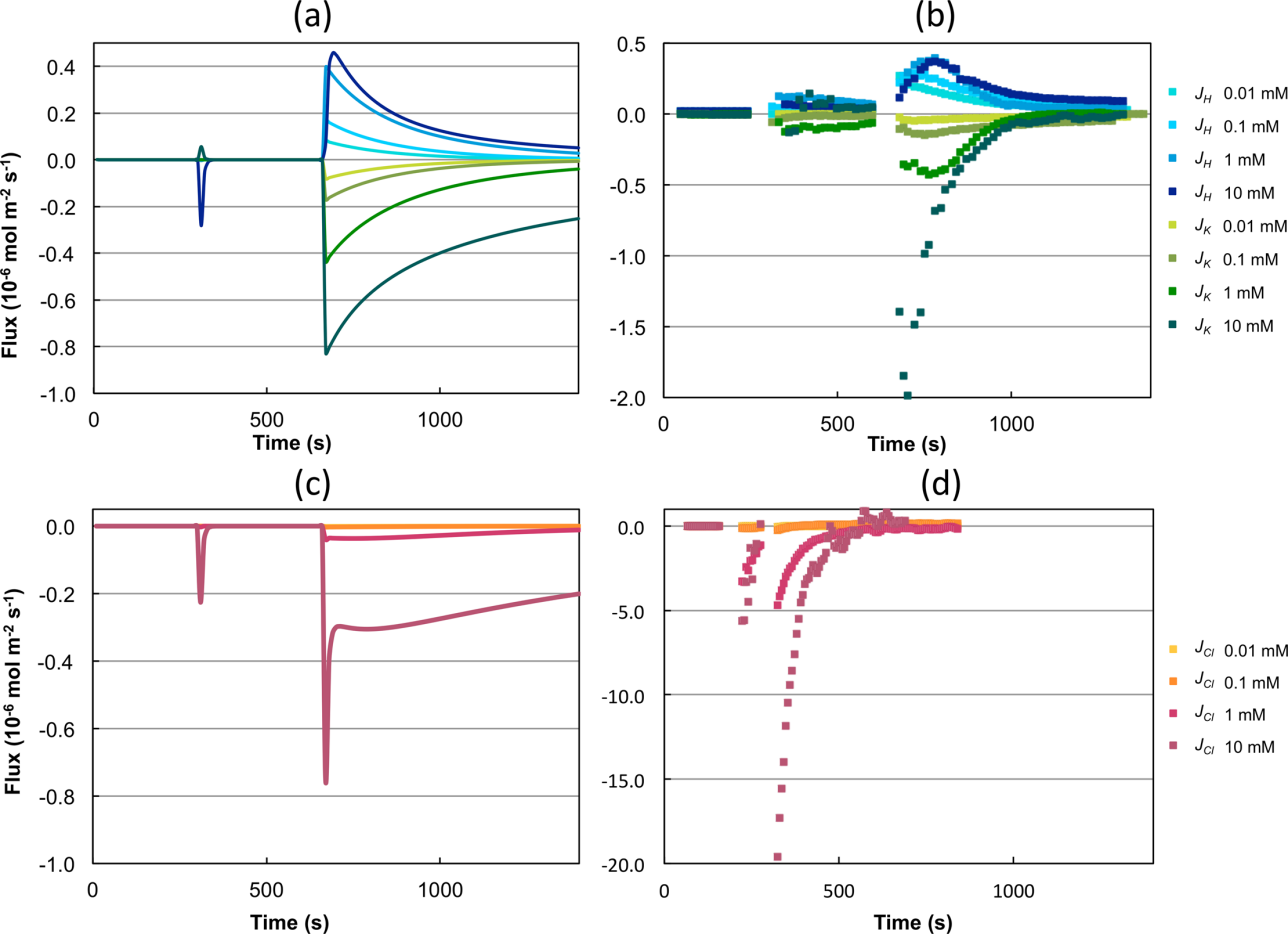
Experimental data and simulation using model 2 with parameter set P2a. The model was used to reproduce the H^+^ and K^+^ flux data from MIFE experiments. It predicted the existence of Cl^-^ fluxes, which were verified in subsequent experiments. (a) Simulation of H^+^ and K^+^ fluxes during four different *in-silico* experiments with addition of KCl (0.01 mM, 0.1 mM, 1 mM, 10 mM) at 300 s followed by glucose addition at 660 s. (b) Experimental data (MIFE) used for fitting the model. (c) Predicted Cl^-^ fluxes during the four simulations. (d) Experimental validation of the existence of Cl^-^ fluxes. Here, KCl (0.01 mM, 0.1 mM, 1 mM, 10 mM) was added at 180 s followed by glucose addition at 300 s. The K^+^ flux is labeled in green, H^+^ flux in blue and Cl^-^ flux in red. Darker colors represent higher KCl concentrations used for the KCl stimulus.

Thereupon the model was fitted with the additional restriction that no K^+^-ATPase exists (*L*_KAr_ = 0). The fitting procedure resulted in different parameter sets giving an equally good fit. In the following, the two best parameter sets, named P2a and P2b, will be described and analyzed. The resulting best fit is shown in Fig 3 produced with parameter set P2a. The fluxes for potassium and protons obtained for the lower added KCl concentrations can be reproduced reasonably well, only the peak at the highest KCl concentration could not be captured appropriately (Fig 3(A) and 3(B)).

The model was used to simulate the flux of Na^+^ and Cl^−^. At the applied initial conditions (internal Na^+^ concentration lower than 30 mM) no Na^+^ fluxes could be obtained by the model. This is a reasonable result since with such marginal internal and external Na^+^-concentrations, Na^+^-fluxes are not to be expected. Instead, the model predicted an influx of Cl^−^ ions, which was maximally pronounced at KCl stimuli of 10 mM (see Fig 3(C)).

To validate the existence of chloride fluxes, a series of experiments using a chloride-sensitive electrode was evaluated. As apparent in Fig 3(D) a considerable influx of Cl^−^ ions can indeed be detected in all four experiments. In addition, the fluxes show the same qualitative behavior as the model predictions but with even higher values, especially for 10 mM.

**Fig 4:**
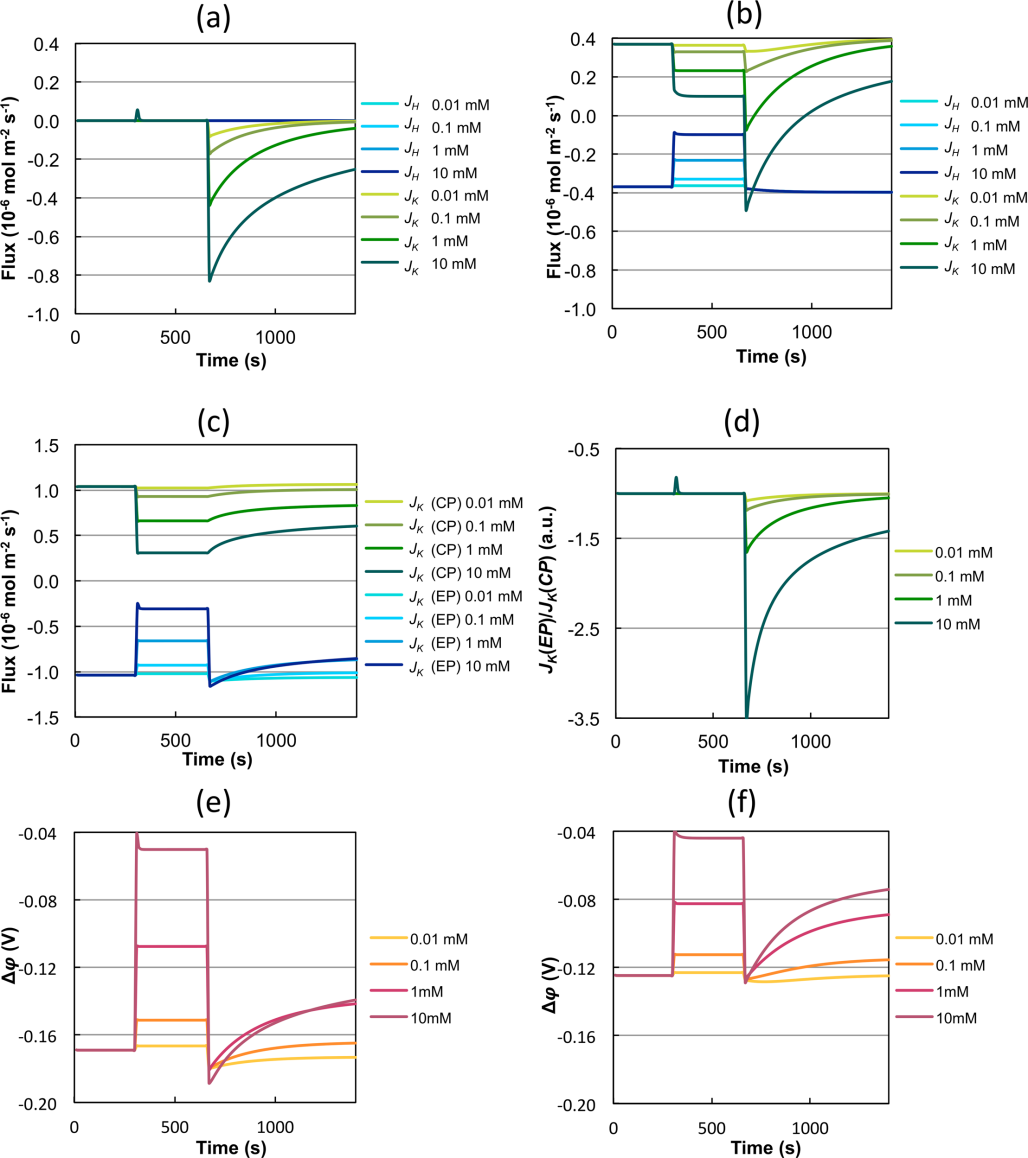
Analysis of individual forces. Separation of *J*_K_ into a K^+^ (green) and a H^+^ (blue) dependent part for simulations with parameter sets a) P2a and b) P2b. c) Separation of *J*_K_ into a chemical (green) and an electrical (blue) potential dependent part with parameter set P2a. d) Ratio of the electrical and the chemical potential dependent part of *J*_K_ with P2a. Simulation of the membrane potential with parameter sets e) P2a and f) P2b.

In simulations using model M2 the membrane potential decreased after glucose addition (shown in Fig 4(E) and 4(F)). This can be initially expected after H^+^ is being pumped out of the cell. Furthermore, model M2 is in agreement with the general assumption that K^+^ enters yeast cells following an activation of the H^+^-ATPase by glucose.

Since the simulations from model M2 appeared to better represent the biological reality we continued using this model for further analyses.

#### Interpretation of phenomenological coefficients in relation to known transporters and directions

The transport processes and reactions characterized by the phenomenological coefficients can be related to the transport proteins and enzymes known for yeast cells. They are listed in Table 2.

The best parameter estimation results from model M2 were analyzed in more detail.

Tables 4 and 5 contain the values for the two sets of estimated parameters and initial conditions that gave the best result. The general model structure, biophysical constants and concentration values taken from literature were the same for both parameter sets; however, they differed in both the estimated parameters and initial conditions. The following section discusses the differences in the phenomenological coefficients.

**Table 4:** Initial concentrations, global quantities and volumes, and estimated parameters for P2a. Estimated model parameters for stress with 4 different concentrations of KCl. All other *L*s could be set to 0 without affecting the goodness of fit.

**Global quantities and volumes**	**Value**	**Source**
*V*_*in*_	1.8· 10^−11^ m^3^	Calculation
*V*_*out*_	2.85 · 10^−6^ m^3^	Exp. condition
*T*	296 K	Exp. condition
*F*	96,485 C/mol	Faraday constant
*Surf* (of all cells)	2.29 · 10^−5^ m^2^	Calculation
Proton buffer capacity (*pbc*)	200 mM pH	Experimental observation
Conversion factor (*cf*)	1000 mM/M	
*K*	1 · 10^−6^	estimated
*C*_*ATP*_	0.316 mM	estimated
Δ*φ*	-0.168 V	estimated
**Initial conditions**	**Values**	**Source**
*H*_*out*_	3.162 ·10^−3^	Exp. condition (pH 5.5)
*K*_*out*_	0.1 mM	Exp. condition
*Cl*_*out*_	0.1 mM	Exp. condition
*ATP*	2.477 mM	estimated between 0 and 2.5 mM
*ATP*_*stimulus*_	2.5 mM	Özalp et al. [65]
*KCl*_*stimulus*_	0.01, 0.1, 1, 10 mM	Exp. condition
pH_*in*_	5.514	estimated between 5 and 7
*K*_*in*_	75.54 mM	estimated between 60 and 100 mM
*Cl*_*in*_	0.545 mM	estimated between 0.1 and 10 mM
*Na*_*in*_	29.98 mM	estimated between 5 and 30 mM
*Na*_*out*_	0.01 mM	estimated between 0.01 and 0.1 mM
**Phenomenological and stoichiometric coefficients**	**Parameter values**	**Source**
*L*_HHinit_	4.8 · 10^−7^ mol^2^/(J· m^2^ · s)	estimated
*L*_HHaG_	5.62 · 10^−1^ mol^2^/(J· m^2^ · s)	estimated
*L*_HNa_	-1.9 · 10^−12^ mol^2^/(J· m^2^ · s)	estimated
*L*_HAraG_	5.79 · 10^−1^ mol^2^/(J· m^2^ · s)	estimated
*L*_HCl_	3.84 · 10^−7^ mol^2^/(J· m^2^ · s)	estimated
*L*_KK_	1.88 · 10^−8^ mol^2^/(J· m^2^ · s)	estimated
*L*_NaNa_	8.98 · 10^−13^ mol^2^/(J· m^2^ · s)	estimated
*L*_ClCl_	3.08 · 10^−7^ mol^2^/(J· m^2^ · s)	estimated
*k*_*incr*HH_	1.05 · 10^−6^ mol^2^/(J· m^2^ · s^2^)	estimated
*k*_*incr*HAr_	1.08 · 10^−6^ mol^2^/(J· m^2^ · s^2^)	estimated
*k*_ATP*incr*_	10 mol/(m^3^ · s)	estimated

**Table 5:** Initial concentrations, global quantities and volumes, and estimated parameters for P2b. Estimated model parameters for stress with 4 different concentrations of KCl. All other *L*s could be set to 0 without affecting the goodness of fit.

**Global quantities and volumes**	**Value**	**Source**
*V*_*in*_	1.8· 10^−11^ m^3^	Calculation
*V*_*out*_	2.85 · 10^−6^ m^3^	Exp. condition
*T*	296 K	Exp. condition
*F*	96,485 C/mol	Faraday constant
*Surf* (of all cells)	2.29 · 10^−5^ m^2^	Calculation
Proton buffer capacity (*pbc*)	200 mM pH	Experimental observation
Conversion factor (*cf*)	1000 mM/M	
*K*	0.294	estimated
*C*_*ATP*_	0.298 mM	estimated
Δ*φ*	-0.124 V	estimated
**Initial conditions**	**Values**	**Source**
*H*_*out*_	3.162 ·10^−3^	Exp. condition (pH 5.5)
*K*_*out*_	0.1 mM	Exp. condition
*Cl*_*out*_	0.1 mM	Exp. condition
*ATP*	2.384 mM	estimated between 0 and 2.5 mM
*ATP*_*stimulus*_	2.5 mM	Özalp et al. [65],
*KCl*_*stimulus*_	0.01, 0.1, 1, 10 mM	Exp. condition
pH_*in*_	5.34	estimated between 5 and 7
*K*_*in*_	99.9 mM	estimated between 60 and 100 mM
*Cl*_*in*_	0.366 mM	estimated between 0.1 and 10 mM
*Na*_*in*_	14.34 mM	estimated between 5 and 30 mM
*Na*_*out*_	0.088 mM	estimated between 0.01 and 0.1 mM
**Phenomenological and stoichiometric coefficients**	**Parameter values**	**Source**
*L*_HHinit_	6.54 · 10^−8^ mol^2^/(J· m^2^ · s)	estimated
*L*_HHaG_	2.39 · 10^−4^ mol^2^/(J· m^2^ · s)	estimated
*L*_HK_	9.79 · 10^−9^ mol^2^/(J· m^2^ · s)	estimated
*L*_HAraG_	1.22 · 10^−4^ mol^2^/(J· m^2^ · s)	estimated
*L*_HCl_	4.51 · 10^−8^ mol^2^/(J· m^2^ · s)	estimated
*L*_KK_	2.2 · 10^−8^ mol^2^/(J· m^2^ · s)	estimated
*L*_ClCl_	3.34 · 10^−8^ mol^2^/(J· m^2^ · s)	estimated
*k*_*incr*HH_	1.63 · 10^−1^ mol^2^/(J· m^2^ · s^2^)	estimated
*k*_*incr*HAr_	8.32 · 10^−2^ mol^2^/(J· m^2^ · s^2^)	estimated
*k*_ATP*incr*_	0.018 mol/(m^3^ · s)	estimated

The most prominent phenomenological coefficients before glucose are *L*_HH_, *L*_HCl_, *L*_KK_, *L*_ClCl_, and *L*_HK_. The first four parameters are unambiguous in the two sets. *L*_HK_, however, exhibits a small negative value in one parameter set, further referred to as P2a, and its deletion does not affect the goodness of fit. In the other parameter set referred to as P2b, it holds a larger positive value, and appears to be of higher relevance.

The most prominent phenomenological coefficients after glucose addition are *L*_HH_ and *L*_HAr_, where the latter can be associated with a change in activity of the Pma1. In P2a the K^+^ flux after glucose is mainly passive *via L*_KK_, whereas the high *L*_HK_ value in P2b indicates coupling with H^+^.

It is of note that the data set used here was obtained for the specific condition of starved cells stimulated with mild KCl concentrations. Thus, we don’t want to exclude the possibility that the other phenomenological couplings can be of strong importance under different conditions.

#### What drives potassium transport through Trk1,2?

The TRK transporters comprise four MPM motifs and evolved by gene duplication and fusion of molecular structures that were originally K^+^ channels. In terms of function it is currently not clear whether Trk1,2 acts as uniporter or cotransports K^+^ together with other ions, e.g. H^+^. The parameter estimation presented above resulted in two alternative parameter sets with either low (P2a) or high (P2b) values of *L*_HK_. A high value of *L*_*HK*_ indicates that transport of K^+^ is directly coupled to H^+^, e.g. *via* symport or antiport. On the contrary, if *L*_HK_ is low, H^+^ could only affect K^+^ indirectly *via* the membrane potential. Since both P2a and P2b resulted in an equally good fit, the present data alone did not allow us to decide, which scenario is more likely. Therefore, we performed additional analyses.

We use the parameter sets P2a and P2b to dissect the contribution of the chemical potentials of K^+^ and H^+^ and the electrical potential to K^+^-flux.

First, the net flux *J*_K_ was separated into the influence due to the electrochemical potentials of the different ions. Since in the model only coupling between K^+^ and H^+^ was considered, *J*_K_ is composed of an H^+^-dependent part

$$J_{\text{K}}\left(\text{H}\right)=L_{\text{KH}}\left(R\;\text{ln}\frac{c_{\text{H}}^{i}}{c_{\text{H}}^{o}}+\frac{F}{T}\Delta \varphi \right)$$

and a K^+^-dependent part

$$J_{\text{K}}\left(\text{K}\right)=L_{\text{KK}}\left(R\;\text{ln}\frac{c_{\text{K}}^{i}}{c_{\text{K}}^{o}}+\frac{F}{T}\Delta \varphi \right)$$

with *J*_K_ = *J*_K_(H) + *J*_K_(K).

Fig 4(A) shows *J*_K_(H) and *J*_K_(K) for P2a. It can be seen that in this case *J*_K_ is exclusively dependent on its own electrochemical potential. It is coupled to gradients of H^+^ only *via* the membrane potential and not directly *via* effects such as symport or antiport. Using P2b (Fig 4(B)) and therefore predicting a K^+^/H^+^ symporter the absolute forces due to K^+^ and H^+^ gradients are roughly equal to each other before the KCl stimulus and also more or less after, although with different intensities depending on the strength of the KCl stimuli. After glucose addition, the shape of the K^+^ dependent part is similar to that shown in Fig 4(A), but much lower and would even result in K^+^-efflux. A higher and positive *L*_HK_ suggesting coupling to the H^+^ gradient by H^+^/K^+^ symport is necessary to explain the K^+^-fluxes observed in the experiment. The flux due to H^+^ gradient stays constant over time, whereas the flux due to K^+^ gradient decreases over time again. The closer the absolute values of K^+^- and H^+^-dependent fluxes, the lower is the net flux *J*_K_ (for comparison see Fig 3).

Next, the dependency of *J*_*K*_ on the forces due to the chemical potential (CP) and the electrical potential (EP) of the ions were analyzed by separating the net flux into a part depending on the chemical potentials

$$J_{\text{K}}\left(\text{CP}\right)=R\left(L_{\text{KH}}\;\text{ln}\frac{c_{\text{H}}^{i}}{c_{\text{H}}^{o}}+L_{\text{KK}}\;\text{ln}\frac{c_{\text{K}}^{i}}{c_{\text{K}}^{o}}\right)$$

and a part depending on the electrical potentials

$$J_{\text{K}}\left(\text{EP}\right)=\left(L_{\text{KH}}+L_{\text{KK}}\right)\frac{F}{T}\Delta \varphi$$

with $J_{\text{K}}=J_{\text{K}}\left(\text{CP}\right)+J_{\text{K}}\left(\text{EP}\right)$.

Although the models using P2a and P2b show distinct ion dependency, they exhibit a similar dependency on the chemical and the electrical potentials. Fig 4(C) shows *J*_K_(CP) and *J*_K_(EP) and Fig 4(D) the quotient *J*_K_(EP)/*J*_K_(CP). Before and after the KCl stimulus, the positive *J*_K_(CP) and the negative *J*_K_(EP) balance each other resulting in a *J*_K_ net flux close to zero (see Fig 3). After the addition of glucose K^+^-efflux driven by the chemical potential is slowly increasing but influx due to the electrical potential is dominating. Directly after the addition of 10 mM KCl and glucose, approximately 3.5 times more K^+^ is imported driven by the electrical potential than exported due to chemical potential. This results in a net influx of K^+^. During the long run, the absolute values of *J*_K_(CP) and *J*_K_(EP) approach each other and, thereby, move towards a new steady state. Fig 4(E) and 4(F) show predictions of the membrane potential using P2a and P2b, respectively. In case of P2a (only K^+^-dependent K^+^-transport) the membrane potential would possess a lower value and changes after addition of KCl and glucose would be more extreme.

#### Prediction of the effect of multiple KCl stimuli with the model

**Fig 5:**
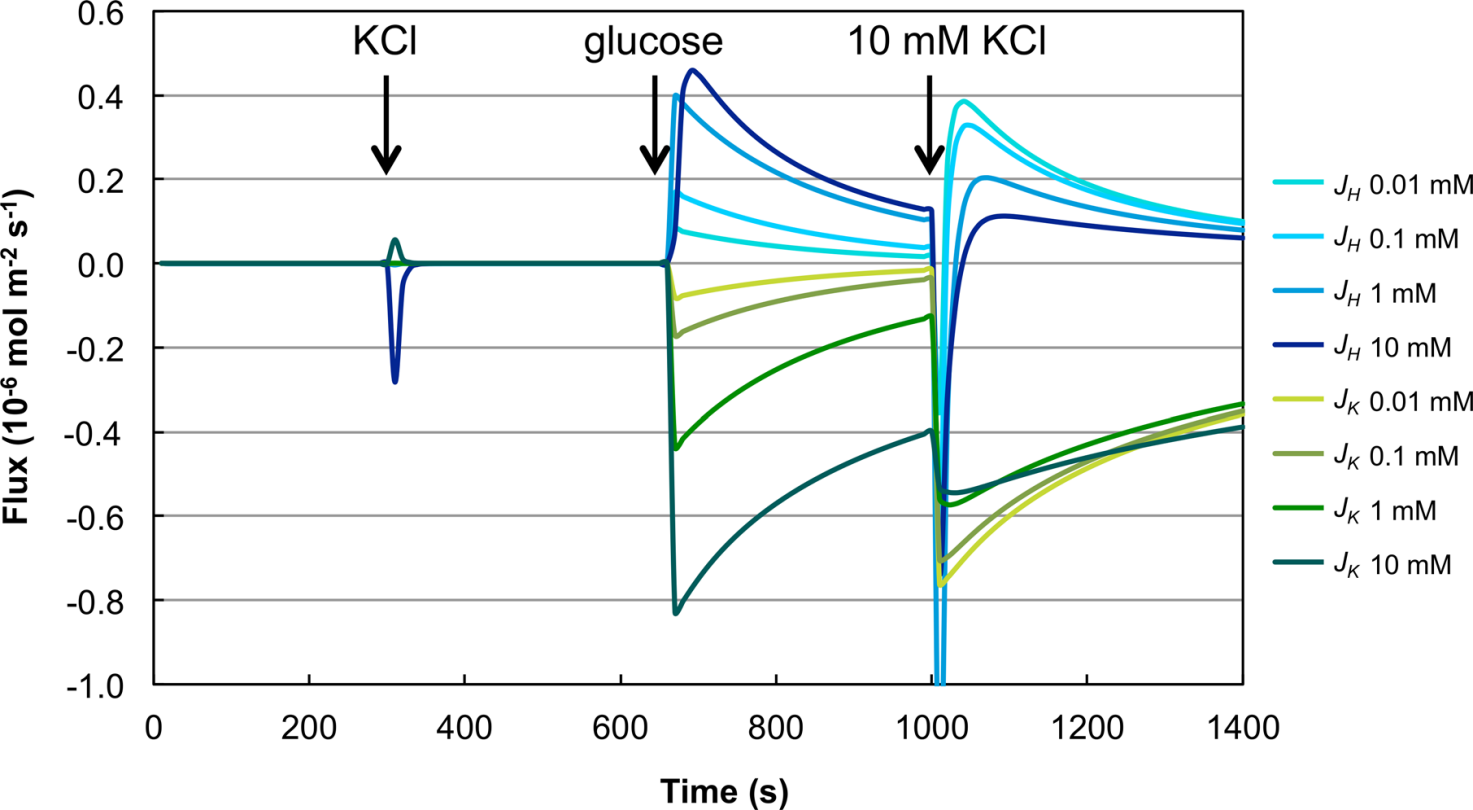
Prediction of second KCl stimulus. Model M2 with parameter set P2a was used to predict the reaction of the system to a second KCl stimulus following the glucose stimulus. As first stimulus the KCl concentrations 0.01, 0.1, 1 and 10 mM were used, in consistency with the data used for model fitting. The second stimulus was modeled as additional 10 mM KCl in all cases. The K^+^ flux is labeled in green, H^+^ flux in blue. Darker colors represent higher KCl concentrations used for the first KCl stimulus (applied to the system prior to time point 0). Glucose was added in this experiment at time point 660 s, the second KCl stimulus was at 1000 s.

We further tested whether and how the yeast cells respond to successive salt stresses. To simulate this case *in silico* P2a was used to predict the effect of a second KCl stimulus of 10 mM at time point 1000 s. The resulting time courses for the H^+^ and K^+^ fluxes are presented in Fig 5. Under all tested conditions (the four different KCl concentrations for the primary stimulus) the model responded to the second KCl stimulus. Although the second KCl stimulus was 10 mM in all experiments, different primary KCl stimuli caused different responses to the second stimulus. Higher initial KCl stimuli led to higher fluxes after glucose addition and to lower fluxes after the second KCl stimulus. At the two highest KCl stimuli a transient H^+^ influx was observed shortly after the second KCl addition.

## Discussion

We introduced a general thermodynamic model for the regulation of ion fluxes through the yeast cellular membrane. This model is based on the acting forces–the electrochemical potentials of the ions–and their interrelations. Using a linear approach we expressed the resulting fluxes without taking into account precise knowledge about the involved channels and transporters.

We restricted the model in its application here to the fluxes of the major cations H^+^, K^+^, Na^+^ and of the anion Cl^−^, the conversion of ATP to ADP as an active driving force, the calculation of the internal pH as well as the change in the membrane potential. This was based on the specific experimental scenario analyzed here. Such conditions enable to measure fluxes of protons, potassium, and chloride. However, the theoretical approach presented here should also be applicable to more complex situations with further ions involved. The systematic thermodynamic formulation of the major components contributing to the maintenance of a stable intracellular cation content may become well suited for the purpose of modeling this complex system, particularly at the current early stage of understanding.

An approach used by others [8,23,41] is to model each transporter or channel separately in great detail. We refrained from doing so due to the unavailability of suitable data that describe the contribution of each individual component to measured overall fluxes. Furthermore, detailed modeling of individual transport reactions increases the complexity of the model and a massive amount of parameters must be estimated or taken from sources in which the experimental conditions might not be comparable with those conditions used here.

The entirely phenomenological approach applied here does not depend on a detailed understanding and description of structure, function, molecular details, or kinetic parameters of individual constituent as parts of the system. Instead, a level of complexity was chosen which is in accordance with the availability of data for net ion flux measurements obtained under physiologically relevant conditions.

By identification of the generalized forces that are responsible for the flux of a given ion, the model is able to assist reinterpreting classical findings on ion flux propagation and provides directions for further efforts aimed at defining transport processes at the molecular level.

The results of the simulations are in good agreement with the experimental observations and the theoretical predictions achieved for the values of the phenomenological coefficients are reasonable from the biological point of view.

For example, the predicted and validated Cl^-^ influx in addition to the H^+^ efflux and K^+^ influx is a reasonable feature from the biological perspective. Since the proton efflux does not reach the same magnitude as the potassium influx, electroneutrality must be ensured by another charged ion. Due to the nature of the experiment (addition of potassium chloride), chloride is available and its influx can compensate the flow of charges by potassium.

The chloride flux is likely to affect the membrane potential and, as predicted by the model, counteract the excess of charge, which would normally build up caused by the asymmetry of the H^+^ and K^+^. At the applied experimental conditions, no Na^+^ fluxes were obtained during the simulation. However, it is also possible that other ions can affect the membrane potential, which are not yet included in the model (e.g. bicarbonate [8] and phosphate [42]) and for which no experimental data were available under the present conditions. As a future perspective, it could be very interesting to consider e.g. recent work on the two main high–affinity phosphate transporters, Pho84 and Pho89 [43,44] for further improvement of the model.

In any case the model is still amenable to development in view of a more comprehensive picture of cation homeostasis. Perspectives and weaknesses of the approach will be discussed as follows.

First of all, the restriction set on the system is that it acts close to equilibrium, which is a prerequisite for the linear approach to hold, and thus that fluctuations are insignificant. This implies certain limitations on the processes. If the gradients of the intensive parameters within the system are large it might not satisfy these requirements. The range of applicability of this theory cannot be specified on *a priori* grounds, and the justification of its use rests, eventually, on the validity of the results obtained.

Furthermore, in the model the distribution of substances in the internal as well as the external volume are assumed to be homogeneous. Although this assumption was used previously [8,45,46], it might be useful to analyze the effect of spatial gradients in future models.

Some intracellular transporters have only recently been identified and characterized. These comprise mainly alkali-metal cation/H^+^- antiporter, located in the vacuolar membrane (Vnx1), [47] endosomal membrane (Nhx1) [48] and the Golgi apparatus membrane (Kha1) [49]. These organellar systems also serve to regulate the intracellular K^+^ — and pH-homeostasis and may play an important role in detoxification of sodium by sequestration in the vacuole. For these intracellular transport systems almost no time resolved biochemical transport data are currently available and were thus not included in the presented model.

A description of the temporal behavior should in general also incorporate the rates of changes of the cell volume due to effects on the intracellular osmolarity and changes of the permeabilities for the ions over time [50–53]. These terms would, in turn, simultaneously affect the values of intracellular cation concentrations [50,54]. Here, the volume was assumed to remain constant during the simulation. This is a reasonable assumption since the concentrations used in the experiments are far below any critical value (experiments studying the osmotic stress response *via* the activation of the Hog-pathway usually start with concentrations of several hundreds of mM NaCl [55–57]) and already at 0.05 M the Hog activation is down to a tenth of the maximum amplitude [58]. Therefore, it is highly unlikely that salt concentrations lower than 0.01 M induce any significant osmotic or volume effects. On the other hand substantial progress has already been made in the field of modeling response to osmotic stress *via* volume and turgor regulation in the yeast *S*. *cerevisiae* [59–61] and both models could highly benefit by getting joined. For further and extended versions of the presented model, a combined observation of the regulation of the osmotic response as well as the homeostasis of the major cations Na^+^, K^+^ and intracellular pH should be envisaged for a broader understanding. As a second future perspective the model should also be validated with the support of proper deletion mutants lacking specific transport systems. The impact of such mutant data would provide insights on the reliability of the model when it was confronted with actual measurements.

## Materials and Methods

### MIFE

Data acquisition was performed by using monolayers of *S*. *cerevisiae* cells (grown in YNB-F supplemented with 50 mM KCl till late-log phase, harvested by centrifugation and washed twice with double-distilled water) immobilized on poly-L-lysine treated glass coverslips. Each cover slip was placed in a total of 3 ml sample buffer volume in a Petri dish. After addition of the specific concentration of KCl the cells were energized with glucose to enable generation of ATP and thus the performance of secondary active transport mechanisms. Net fluxes of K^+^ and H^+^ were measured non-invasively using the microelectrode ion flux measuring (MIFE; University of Tasmania, Hobart, Australia) technique as described by Shabala *et al*. [62] [63]

### Initial values and parameters

The surface of all cells, *Surf* and the inner volume, *V*_*in*_ were calculated from the detected optical density (1.2 · 10^7^ cells per ml OD 1) applied to achieve a cell monolayer and by assuming a single cellular surface of 63,6 μm^2^ (based on a round cell with a diameter of 4.5 μm) and a volume of 50 fL according to [64]. The value for V_out_ was obtained directly from the experimental setup.

ATP was estimated to be between 0 and 2.5 mM ahead of the glucose addition. The available ATP after the glucose stimulus was supposed to reach 2.5 mM, according to previous observations by [65], and described respectively in Eq 11. It was assumed that in the starved cells no ATP is available for other than basic vital processes and that addition of glucose is necessary to induce primary and secondary active transport mechanisms [35,36]. Accordingly, the parameters *L*_iAr_ (*i* ϵ {H,K}) were initially set to 0. It was assumed that only those parameters directly or indirectly involved in primary active transport *L*_iAr_ (*i* ϵ {H,K}) and *L*_*ii*_ (*i* ϵ {H,K}) can change after glucose and that the Onsager relation holds.

### Time course simulation and parameter estimation

The model implementation, time course simulation and parameter estimation were performed in COPASI [66]. COPASI comes with a set of implemented optimization methods, which can be used to estimated parameters and initial conditions of mathematical models. Of the given methods, the particle swarm optimization method gave the best results for the model at hand. The particle swarm optimization method [67] imitates the behavior of a biological swarm (e.g. a flock of birds) to iteratively optimize model parameters. Starting with given parameter values, the method searches through the parameter space to find the optimal parameter set, i.e. the parameter set which minimizes the error between the current model solution and the experimental values. For this, each parameter set has a position and velocity in the parameter space and also remembers its best-achieved value and position. Depending on its own information and the position of its neighbors a new velocity is calculated and the parameters are updated. More information about the implementation of the algorithm in COPASI can be found at http://www.copasi.org.

To minimize the problem of being trapped in local minima, a Python script was implemented to run the particle swarm algorithm 1000 times with random initial parameter values as well as random upper and lower parameter bounds. For the estimation of the initial conditions experimentally verified concentration ranges were used (see Tables 3–5). The “straight coefficients" *L*_*ii*_ were allowed to be positive only, the “cross coefficients" *L*_*ij*_ were allowed to be either positive or negative. The options iteration limit 400, swarm size 40, standard deviation 1e−6, random number generator Mersenne Twister [68] and random seed showed good results at a reasonable duration. The best matching parameter sets of the 1000 runs were finally taken; in case a parameter was located at a boundary, this boundary was extended by a factor of 100 and subsequent parameter estimation was performed. The time course simulation was solved with the deterministic LSODA method [69].

## Supporting Information

S1 Text This file contains a description of the sensitivity analysis and of the behavior of phenomenological coefficients over time. (DOCX)

S1 Data Data representing net fluxes of K^+^ and H^+^ measured non-invasively using the microelectrode ion flux measuring (MIFE; University of Tasmania, Hobart, Australia) technique as described in the section Materials and Methods. Fluxes have been measured over time after addition of 10μM, 100μM, 1mM, or 10mM KCl to cells of *S*. *cerevisiae*.
(XLSX)
