# Supplementary material for: A Thermodynamic Model of Monovalent Cation Homeostasis in the Yeast Saccharomyces cerevisiae
Source: PLoS Comput Biol. 2016 Jan 27;12(1):e1004703. doi: 10.1371/journal.pcbi.1004703 (PMC4729481; doi:10.1371/journal.pcbi.1004703)
Supplement: S1 Text — (DOCX) [file pcbi.1004703.s001.docx]

**Supplementary Material for**

**A Thermodynamic Model of Monovalent Cation Homeostasis in the yeast *Saccharomyces cerevisiae***

**Susanne Gerber** 1,2,6**, Martina Fröhlich** 2,5,6**, Hella Lichtenberg-Fraté** 3**, Sergey Shabala** 4**, Lana Shabala** 4**, and Edda Klipp** 2*

^1^Faculty of Biology, Johannes Gutenberg-Universität Mainz, Germany,

^2^Theoretical Biophysics, Humboldt-Universität zu Berlin, Germany,

^3^Molecular Bioenergetics, University of Bonn, Germany,

^4^School of Land and Food, University of Tasmania, Australia

^5^Babraham Institute, Cambridge, United Kingdom

^6^These authors contributed equally to this work.

* Corresponding author

Contact: [edda.klipp@rz.hu-berlin.de](mailto:edda.klipp@rz,hu-berlin.de)

1. **Sensitivity Analysis**
2. **Behavior of phenomenological coefficients over time**

**1. Sensitivity Analysis**

A sensitivity analysis was performed to identify which model parameters have the highest influence on the occurrence of the chloride flux. Precisely, it was analyzed, how slight parameter changes affect the maximal chloride flux. To this end, we neglected the sharp changes directly after the change of the stimulus and set our focus on the smooth part of the curve. Since this occurs at different time points for the different models and parameter sets used, sensitivity analysis was also performed at different time points, which is 690 s for P1, 790 s for P2a and 990 s for P2b.

The scaled sensitivity coefficients are presented in Fig. S1.

The results show that the different parameter sets of the model vary also with respect to the parameters the Cl^-^ flux is sensitive to. All 3 parameter sets have in common that Cl^-^ flux is sensitive to the concentration of KCl applied. Furthermore, for all sets, the ATPase function is important, here represented by the concentration of ATP in steady, $\bar{c}_{ATP}$. However, they differ in their relevance for the phenomenological coefficient. Cl^-^ in P1 is most sensitive to the parameters involved in the dynamics of the K^+^-ATPase. In P2a and P2b, Cl^-^ is sensitive to different parts of Cl^-^ and active H^+^ transport. In P2a, a change in the *L*_ClCl_ has the highest effect, whereas in P2b *L*_HCl_ and *L*_ClCl_ are equally involved, suggesting different Cl^-^ transport mechanisms in these two parameter sets. Regarding the ATPase, the main focus is in P2a on the initial dynamics, whereas in P2b it is on the final value.


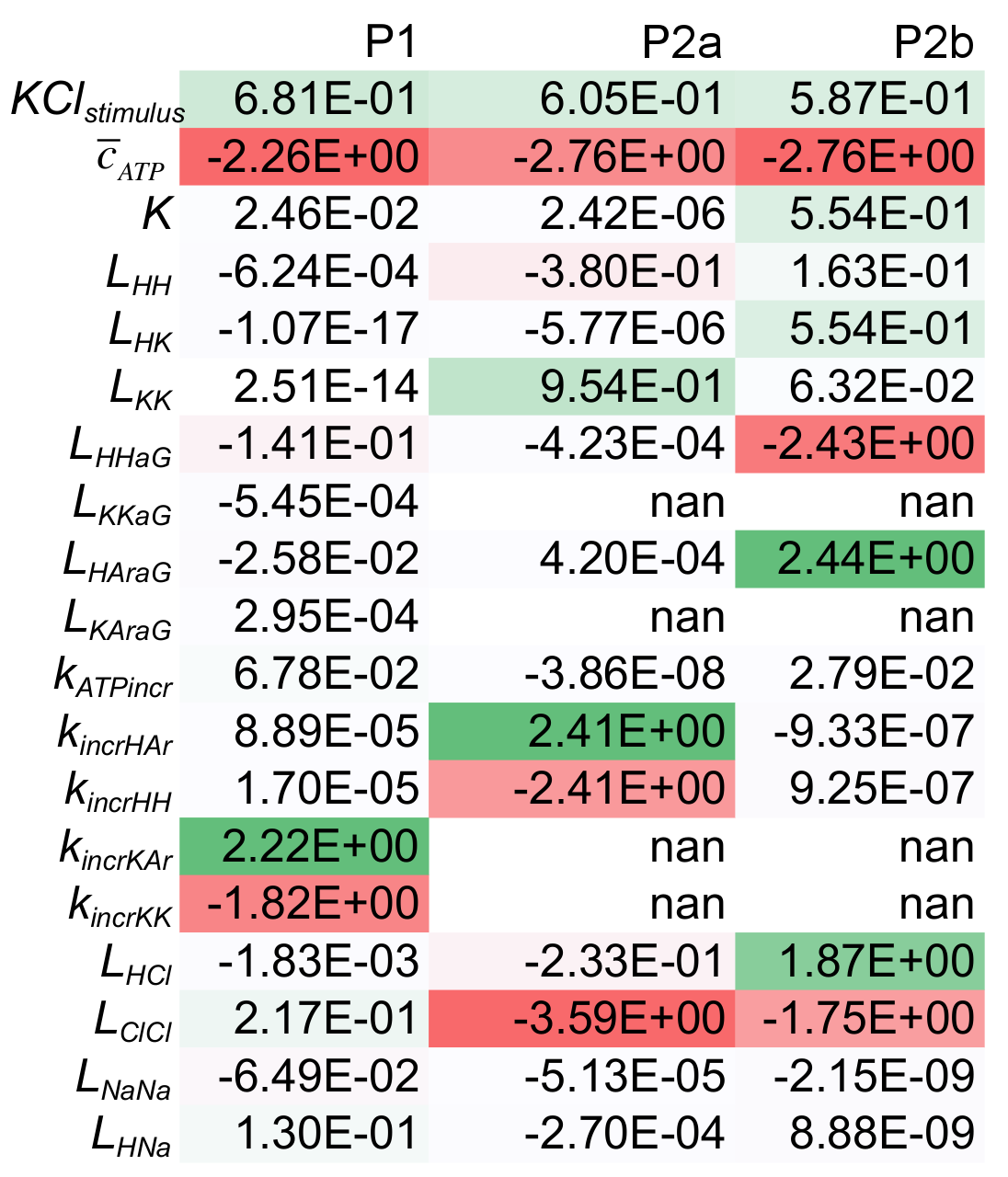


Figure S1: Sensitivity analysis was performed in Copasi. It was analyzed, how small changes in each individual parameter value affect the chloride flux. Sub task “time series” was selected and in the Time series task the respective simulation times were chosen (690 s for P1, 790 s for P2a and 990 s for P2b).

**2. Behavior of phenomenological coefficients over time**

We assumed that the model contains two types of phenomenological coefficients, i.e. for ATP dependent and independent processes. Those, that are not involved in ATP-dependent processes were estimated directly and assumed to remain constant throughout the simulation.

Those, which are involved in ATP-dependent processes, were allowed to change after glucose addition at 660 s. To this end, maximal possible values of the parameters as well as constants for their dynamical behavior were estimated and the values of the coefficients over time were calculated according to Equation 13 in the main text.

During the parameter estimation process, we did not restrict the model in that the maximal values of the parameters need to be reached during the time of the simulation. Thus, we analyzed, until which time the increase of the parameters is necessary to describe the model behavior. P2b reaches its maximum value during the first second after the stimulation. In both P2a and P2b the maximum values are not reached during the time of the simulation. However, further increase of the parameters can be stopped at 800 s for P1 and at 2 s for P2a without affecting the simulation outcome. The behaviors of the resulting phenomenological coefficients during these simulations are presented in Fig. S2.

The values that can change over time are for an H^+^-ATPase *L*_HAr_ and *L*_HH_ (due to backpressure effects of the pump). For P1 these are in addition the values for a potential K^+^-ATPase *L*_KAr_ and *L*_KK_.

In the simulation for P1 *L*_KAr_ and *L*_KK_ are the dominant parameters after glucose. That *L*_KK_ is higher than *L*_KAr_ can happen if the pump transports multiple K^+^ ions per consumption of ATP.

First, it was surprising, that *L*_ClCl_ has such a high value. But, as can be seen in Fig. S1, P1 is quite robust to changes in this parameter.

In both P2a and P2b the highest parameter values are those affecting the H^+^-ATPase. Also here, after glucose addition the values for *L*_HH_ are higher than for *L*_HAr_. However, only in P2b is the increase of *L*_HH_ much stronger than of of *L*_HAr_, suggesting that more H^+^ ions are pumped per ATP consumption. However, in P2a, the increase of *L*_HH_ and *L*_HAr_ are about the same, thus suggesting a more inefficient pump with only 1 H^+^ ion per ATP.


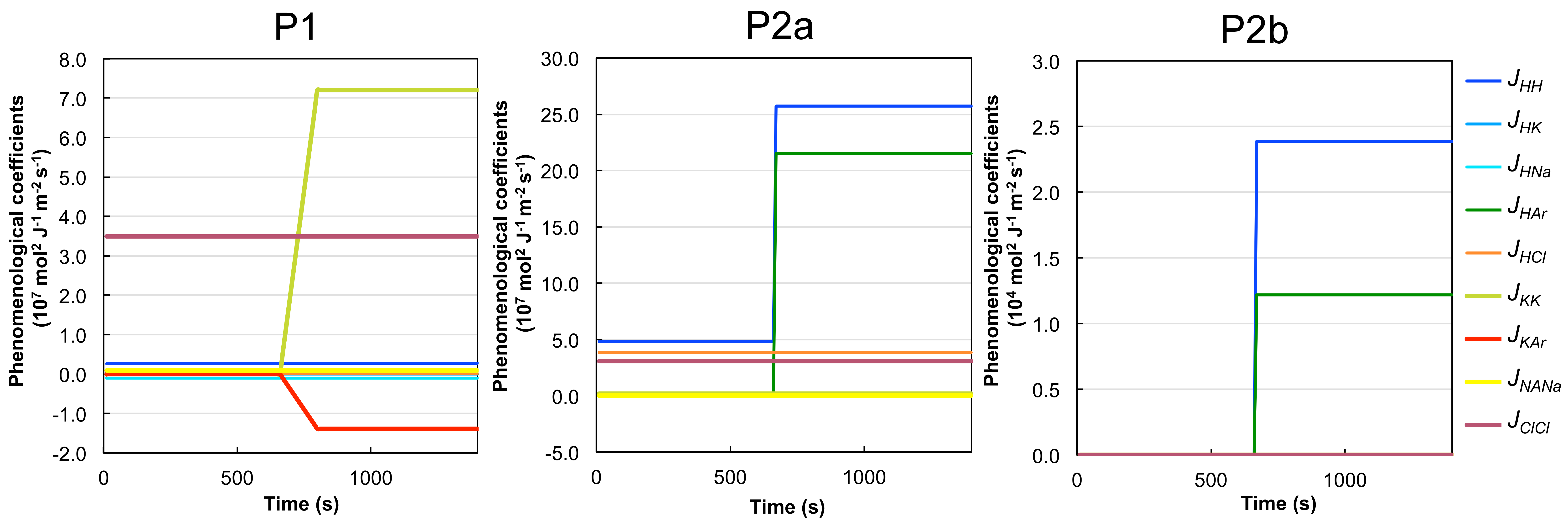


Figure S2: Behavior of the phenomenological coefficients over time. The time point for the glucose stimulus is 660 s.
